# Associations of childhood adversity and substance use disorder polygenic scores with disorder severity and diagnostic criteria

**DOI:** 10.1017/S0033291725001163

**Published:** 2025-05-02

**Authors:** Jackson F. SooHoo, Christal N. Davis, Angela Han, Zeal Jinwala, Joel Gelernter, Richard Feinn, Henry R. Kranzler

**Affiliations:** 1 Center for Studies of Addiction, Department of Psychiatry, University of Pennsylvania School of Medicine, Philadelphia, PA, USA; 2 Mental Illness Research, Education, and Clinical Center, Crescenz VAMC, Philadelphia, PA, USA; 3 Department of Psychiatry, Yale University School of Medicine, New Haven, CT, USA; 4 VA Connecticut Healthcare System, West Haven, CT, USA; 5 Department of Medical Sciences, Frank H. Netter School of Medicine, Quinnipiac University, North Haven, CT, USA

**Keywords:** Childhood adversity, polygenic scores, substance use disorders, diagnostic criteria, gene-environment interaction

## Abstract

**Background:**

Genetic and environmental factors, including adverse childhood experiences (ACEs), contribute to substance use disorders (SUDs). However, the interactions between these factors are poorly understood.

**Methods:**

We examined associations between SUD polygenic scores (PGSs), ACEs, and the initiation of use and severity of alcohol (AUD), opioid use disorder (OUD), and cannabis use disorder (CanUD) in 10,275 individuals (43.5% female, 47.2% African-like ancestry [AFR], and 52.8% European-like ancestry [EUR]). ACEs and SUD severity were modeled as latent factors. We conducted logistic and linear regressions within ancestry groups to examine the associations of ACEs, PGS, and their interaction with substance use initiation and SUD severity.

**Results:**

All three SUD PGS were associated with ACEs in EUR individuals, indicating a gene–environment correlation. Among EUR individuals, only the CanUD PGS was associated with initiating use, whereas ACEs were associated with initiating use of all three substances in both ancestry groups. Additionally, a negative gene-by-environment interaction was identified for opioid initiation in EUR individuals. ACEs were associated with all three SUD severity latent factors in EUR individuals and with AUD and CanUD severity in AFR individuals. PGS were associated with AUD severity in both ancestry groups and with CanUD severity in AFR individuals. Gene-by-environment interactions were identified for AUD and CanUD severity among EUR individuals.

**Conclusions:**

Findings highlight the roles of ACEs and polygenic risk in substance use initiation and SUD severity. Gene-by-environment interactions implicate ACEs as moderators of genetic susceptibility, reinforcing the importance of considering both genetic and environmental influences on SUD risk.

## Introduction

Substance use disorders (SUDs) are chronic conditions characterized by impaired control over substance use and disrupted functioning. In 2022, SUDs affected more than 48 million U.S. individuals (Center for Behavioral Health Statistics and Quality, 2023), contributing to morbidity and mortality (Ahmad, Cisewsk, Rossen, & Sutton, 2024; National Institute on Alcohol Abuse and Alcoholism (NIAAA), 2024). Despite their prevalence and impact, there are gaps in our understanding of SUDs, including how known risk factors, such as genetic liability and adverse childhood experiences (ACEs), may differentially relate to substance use initiation and SUDs.

Genetic, environmental, and gene-by-environment influences are all associated with the development of SUDs (Deak & Johnson, 2021; Milaniak, Watson, & Jaffee, 2015). Although influenced by environmental factors (particularly family environment), SUDs are highly polygenic, with heritability estimates of about 50% (Deak & Johnson, 2021). Compared to SUDs, twin studies show that substance use initiation is more strongly influenced by environmental factors, with heritability estimates of only about 40% (Agrawal & Lynskey, 2006; Kendler, Karkowski, Neale, & Prescott, 2000; Koopmans, Slutske, van Baal, & Boomsma, 1999; Verweij et al., 2010). In addition, twin and molecular genetic studies suggest a combination of shared and distinct genetic and environmental factors influencing substance use initiation and the development of the disorder. For example, twin studies have found that 62% of the genetic variation in cannabis abuse is shared with initiation, with the remaining proportion being genetic influences unique to abuse (Gillespie, Neale, & Kendler, 2009). Similarly, recent genome-wide association studies (GWASs) of substance use have demonstrated that genetic liability for initiation/use and disorder are genetically correlated (Johnson et al., 2020; Quach et al., 2020; Sanchez-Roige, Palmer, & Clarke, 2020). However, initiation and disorder also have some distinct genetic correlations, with these associations sometimes being in opposite directions (Pasman et al., 2018).

Polygenic scores (PGSs) can provide an aggregate estimate of an individual’s genetic liability for SUDs, capturing the polygenic nature of substance use initiation and SUDs. Prior research using phenome-wide association studies (PheWAS) showed that substance-related PGS are associated with substance use initiation and SUDs, as well as other related traits (e.g., major depressive disorder criterion count, PTSD criterion count, and health rating; Kember et al., 2023). With the availability of more powerful GWAS, it is critical to further examine these associations and consider the potential role of moderating environmental variables.

ACEs, which have been associated with risk for SUDs and other psychiatric disorders (Leza, Siria, López-Goñi, & Fernández-Montalvo, 2021), may interact with genetic liability to influence both initiation of use and the severity of SUDs. ACEs include physical, sexual, and emotional abuse; neglect; exposure to violence; and familial instability. Although common in the general population (57.6%) (Madigan et al., 2025), ACEs are substantially more common among individuals with SUDs, with estimates that 85.4% to 100% of individuals in SUD treatment have experienced at least one ACE (Leza et al., 2021). A possible mechanism for this association is that individuals may use substances to cope with the trauma of ACEs, which often adversely affect emotion regulation and contribute to the development of maladaptive coping strategies (Cloitre et al., 2009). Consistent with this, a previous study found that individuals who experienced more ACEs were more likely to develop mood and anxiety disorders than those who had not, and these disorders in turn contributed to increased risk of developing SUDs (Kranzler et al., 2024). Prior studies have not, however, examined the interaction between PGS and ACEs in relation to substance use initiation and SUD severity, leaving gaps in our understanding of how these factors jointly shape risk across the continuum from initiation to disorder.

The current study builds upon previous work that examined the association of ACEs and genetic risk with SUDs (Kranzler et al., 2024; Leza et al., 2021; Meadows et al., 2023; Meyers et al., 2015) by examining: (1) gene–environment correlations (rGE) between PGS and ACEs, (2) associations of ACEs and PGS with substance use initiation and latent AUD, OUD, and cannabis use disorders (CanUDs) severity, and (3) gene-by-environment (i.e., PGS × ACEs) interactions on substance use initiation and SUD severity. We expected ACEs to demonstrate more consistent associations with both substance use initiation and SUD severity than PGS. By examining these associations, this study offers a more comprehensive understanding of the interplay among genetic liability, childhood adversity, substance use initiation, and SUDs.

## Methods

### Participants

Participants were 10,275 individuals from the Yale-Penn sample, a small nuclear family and case–control sample ascertained for studies of the genetics of SUDs. Participants were recruited at Yale University, UConn Health, the University of Pennsylvania, the Medical University of South Carolina, and McLean Hospital. Recruitment was from addiction treatment centers, psychiatric services, and through advertisements (Gelernter et al., 2014). Participants gave written informed consent, and study procedures were approved by institutional review boards at all five sites.

The sample comprised nearly equal numbers of self-identified Black/African American (N = 4,311) and White (N = 4,652) individuals, and smaller numbers of individuals of Hispanic/Latino ethnicity (N = 656) and other race/ethnic groups (N = 656). Based on genetic principal component (PC) analysis, 4,851 individuals were estimated to be of African-like ancestry and 5,424 individuals of European-like ancestry (Table 1).

**Table 1.** Sample characteristics (N = 10,275)

Characteristic	Prevalence (N) or Mean ± SD
*Sex*	
Male	56.20% (n = 5,775)
Female	43.80% (n = 4,500)
*Age*	40.59 ± 11.72
*Genetic ancestry*	
European-like	52.79% (n = 5,424)
African-like	47.21% (n = 4,851)
*Self-reported race*	
White	48.96% (n = 5,030)
Black/African American	44.64% (n = 4,587)
Other	6.40% (n = 658)
* Married*	56.89% (n = 5,846)
* Education (yr)*	12.78 ± 3.04
* Employed*	57.50% (n = 5,908)
*Substance dependence*	
Alcohol	65.64% (n = 6,744)
Cannabis	42.94% (n = 4,412)
Opioids	32.65% (n = 3,355)

*Note:* SD, standard deviation.

### Measures

Participants completed the Semi-Structured Assessment for Drug Dependence and Alcoholism (SSADDA) (Pierucci-Lagha et al., 2007), an interview that assesses lifetime DSM-IV and DSM-5 SUD and psychiatric disorder diagnostic criteria, including for AUD, OUD, and CanUD. Prior to assessing SUD diagnostic criteria, individuals were asked if they had ever used the substance. Those who reported never having used a substance were excluded from SUD analyses for that substance, resulting in a sample of cases and exposed controls for the SUD severity models. As a sensitivity analysis, we used an alternative coding to include both exposed and unexposed controls (see Supplementary Materials).

The SSADDA also assesses demographics (e.g., age, sex, relationship/marital status, education, and annual income) and environmental characteristics, including ACEs. Ten variables were used to assess childhood adversity: two reflecting an unstable home life, three measuring traumatic experiences (physical or sexual abuse or witnessing/experiencing a violent crime), two measuring substance use within the household, and three measuring protective factors. These ten variables were used to derive a latent factor representing ACEs (AFR: CFA = .997, RMSEA = .008, SRMR = .057; EUR: CFA = .954, RMSEA = .030, SRMR = .059), with item loadings that ranged from 0.128 to 0.775 (Supplementary Table 1). Although some item loadings were modest, all were significant, and the factor structure was supported by the overall model fit. This 10-item factor has also previously been used within the Yale-Penn sample to explore the role of ACEs in SUD-related outcomes, providing a foundation for its use in the current study (Kranzler et al., 2024).

In addition to completing the SSADDA, participants provided blood or saliva samples for DNA extraction and genotyping (Gelernter et al., 2014). Genotyping was conducted using the Illumina HumanCoreExome array, Illumina HumanOmni1-Quad microarray, or the Illumina Multi-Ethnic Global array (Illumina, Inc.). We used the Michigan Imputation Server to impute the genetic data with the 1000 Genomes Project Phase 3 as the reference panel (Das et al., 2016). Genetic ancestry was inferred by calculating PCs using single-nucleotide polymorphisms (SNPs) common to both Yale-Penn and the 1000 Genomes Phase 3 reference panel in PLINK 1.9. Individuals were assigned to the closest population group based on distances from 10 PCs (Kember et al., 2023).

Prior to generating PGS, we obtained summary statistics from GWAS for AUD (N_AFR_ = 121,710, N_EUR_ = 751,607; Zhou et al., 2023), OUD (N_AFR_ = 88,498, N_EUR_ = 302,585; Kember et al., 2022), and CanUD (N_AFR_ = 122,271, N_EUR_ = 884,244; Levey et al., 2023) (Supplementary Table 2). In GWAS that included the Yale-Penn sample, we used summary statistics that excluded that sample to ensure the target sample’s independence. For each GWAS, we used ancestry-specific summary statistics and an ancestry-matched linkage disequilibrium (LD) reference panel to calculate PGS. Using PRS-CS software, which uses a Bayesian approach that leverages continuous shrinkage priors to adjust SNP effect sizes while preserving informative signals and accounting for LD structure (Ge et al., 2019), we calculated PGS in the Yale-Penn sample. We applied the software’s “auto” option to estimate shrinkage parameters, with the random seed set to one.

### Data analysis

We generated latent variables reflecting ACEs and AUD, OUD, and CanUD severity using confirmatory factor analysis and the weighted least square mean and variance-adjusted estimator in Mplus v8.11 (Muthén & Muthén, 1998–2017). To validate the SUD PGS, we first examined their associations with SUD diagnoses in the Yale-Penn sample using within-ancestry logistic regression models that included age, sex, and the first 10 genetic PCs as covariates. In addition, we assessed the presence of gene–environment correlations (*rGE*) by calculating Pearson correlations between each PGS and the ACEs factor.

Next, we examined the association between the PGS, ACEs factor, and initiation of use of alcohol, opioids, and cannabis using logistic regression models. We then conducted linear regression models to examine the associations of the PGS and ACEs factor with AUD, OUD, and CanUD severity. Gene-by-environment interactions between PGS and ACEs were estimated in the same models as the main effects for both initiation and SUD severity. To reduce the potential for confounding, all regression models controlled for age, sex, PGS×age, PGS×sex, ACEs×age, ACEs×sex, and the first ten within-ancestry genetic PCs. Significant gene-by-environment interactions were explored using Johnson–Neyman plots to identify the regions of significance, or specifically the range of PGS at which the association between ACEs and the outcome was statistically significant. To examine the impact of limiting SUD severity models to exposed individuals, we conducted sensitivity analyses in which we also included unexposed (never users) individuals.

## Results

### PGS validation and gene–environment correlations

The population-specific AUD and CanUD PGS were associated with their respective DSM-5 SUD diagnoses in EUR individuals (AUD: b = 0.261, SE = 0.058, p < 0.001; CanUD: b = 0.107, SE = 0.049, p = 0.029). The AUD PGS was associated with a DSM-5 AUD diagnosis in AFR individuals (b = 0.141, SE = 0.066, p = 0.032). PGS were higher among individuals with SUDs than those without SUD diagnoses in both the EUR (AUD: M_cases_ = 0.137, M_controls_ = −0.193; OUD: M_cases_ = 0.076, M_controls_ = −0.095; CanUD: M_cases_ = 0.156, M_controls_ = 0.004) and AFR groups (AUD: M_cases_ = 0.039, M_controls_ = −0.101; OUD: M_cases_ = 0.101, M_controls_ = −0.024; CanUD: M_cases_ = 0.024, M_controls_ = 0.013).

In EUR and AFR individuals, there were positive covariances between the CanUD severity factor and the AUD and OUD severity factors (*b* = 0.093–0.359; Table 2). In AFR, but not EUR, individuals, the AUD severity factor covaried with the OUD severity factor *(b =* 0.068, SE = 0.032, p = 0.033). In EUR individuals, ACEs factor scores were correlated with genetic liability for AUD (*rGE* = 0.120, 95% CI = 0.092–0.147, p < 0.001), OUD (*rGE* = 0.164, 95% CI = 1.37–1.90, p < 0.001), and CanUD (*rGE* = 0.192, 95% CI = 0.166–0.219, p < 0.001). In AFR individuals, ACEs were correlated with OUD (*rGE* = 0.032, 95% CI = 0.002–0.061, p = 0.036), but neither AUD (*rGE* = 0.013, 95% CI = −0.017-0.042, p = 0.405) nor CanUD (*rGE* = 0.010, 95% CI = −0.020-0.039, p = 0.517).

**Table 2.** Results of logistic regression models for lifetime substance use initiation

	African-like			European-like		
Model	Estimate	SE	P-value	Estimate	SE	P-Value
*Alcohol use*						
AUD PGS	0.21	0.15	0.15	0.10	0.14	0.46
ACEs	0.92	0.21	<.001	0.84	0.17	<.001
ACEs × AUD PGS	−0.09	0.20	0.66	−0.02	0.16	0.88
*Cannabis use*						
CanUD PGS	0.32	0.05	<.001	0.04	0.05	0.39
ACEs	0.59	0.06	<.001	0.73	0.06	<.001
ACEs × CanUD PGS	−0.05	0.06	0.36	0.02	0.06	0.69
*Opioid use*						
OUD PGS	0.24	0.13	0.07	0.07	0.04	0.07
ACEs	0.58	0.04	<.001	0.33	0.03	<.001
ACEs × OUD PGS	−0.11	0.03	<.001	−0.03	0.03	0.29

*Note:* Models adjusted for first 10 genetic ancestry principal components, sex, age, and the interactions of sex and age with ACEs and PGS. SE, standard error; AUD, alcohol use disorder; PGS, polygenic score; ACEs, adverse childhood experiences factor; CanUD, cannabis use disorder; OUD, opioid use disorder.

### Multivariate regression models

**Figure 1.**
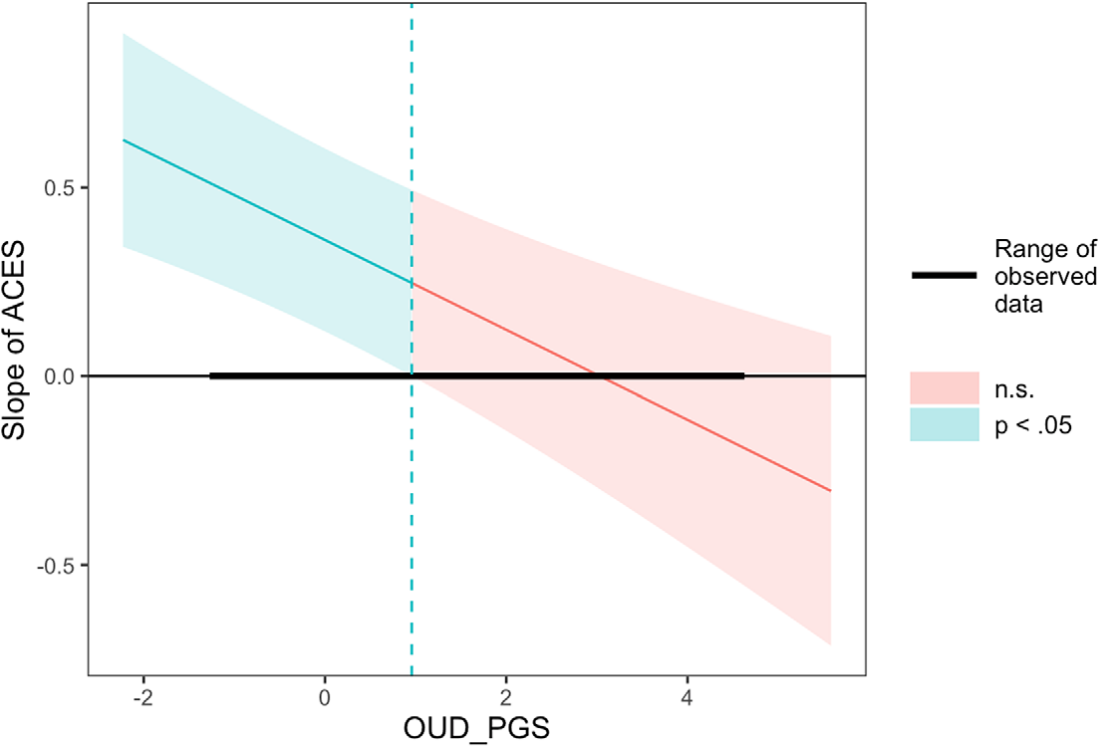
Johnson–Neyman plot of the significant gene-by-environment interaction on opioid use initiation. Note: ACEs, ‘adverse childhood experiences’; OUD, ‘opioid use disorder’; PGS, ‘polygenic score’.

For the models examining associations with substance use initiation, only the CanUD PGS (but not other SUD PGS) was associated with initiating use in EUR individuals (b = 0.315, SE = 0.054, p < 0.001). In contrast, there were positive associations between ACEs and the initiation of all three substances across both ancestries (Table 2). In addition, there was a negative interaction between ACEs and PGS on opioid initiation in EUR individuals (Figure 1). The association of ACEs with opioid initiation decreased as PGS increased and became nonsignificant for PGS 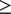 0.96 standard deviations above the mean.

**Figure 2.**
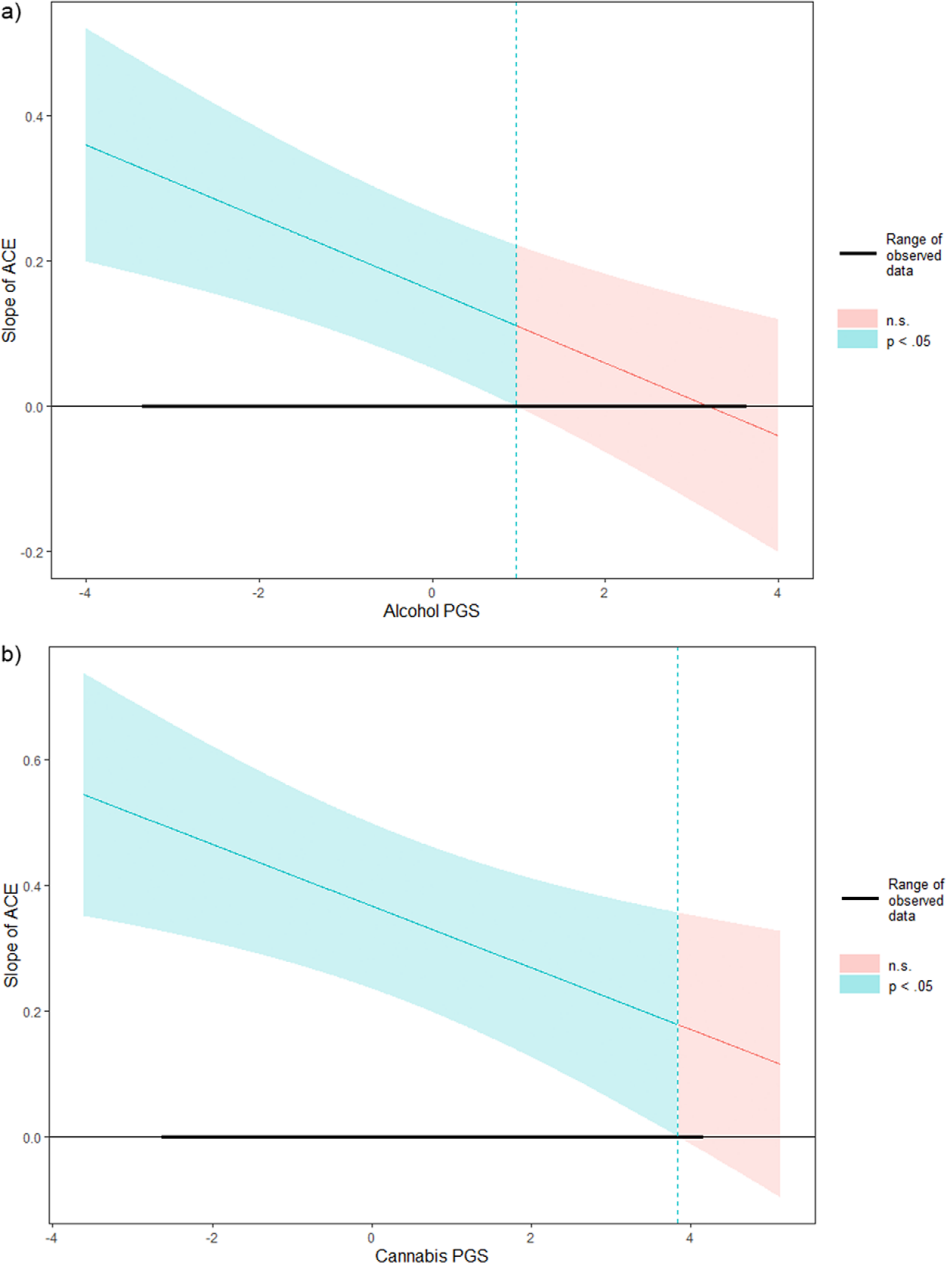
Johnson–Neyman plots of significant gene-by-environment interactions on substance use disorder severity. (a) results for alcohol use disorder severity, (b) results for cannabis use disorder severity. Note: PGS, ‘polygenic score’; ACE, ‘adverse childhood events factor’.

The AUD PGS was not associated with AUD severity in either AFR or EUR individuals. There were positive associations between ACEs and AUD severity in EUR (b = 0.179, SE = 0.052, p = 0.001) and AFR (b = 0.316, SE = 0.058, p < 0.001) individuals (Table 3). There was a negative gene-by-environment interaction (b = −0.049, SE = 0.015, p = 0.001) in EUR, but not AFR, individuals. A Johnson–Neyman plot of the interaction indicated that the association between ACEs and AUD severity in EUR individuals weakened as AUD PGS increased, becoming nonsignificant among those with PGS that were 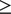 one standard deviation above the mean (Figure 2a).

**Table 3.** Results of linear regression models for substance use disorder severity

	African-like			European-like		
Factor	β	SE	P-value*	β	SE	P-value*
*Alcohol*						
AUD PGS	.030	.064	.639 (.767)	.-.012	.039	.748 (.748)
ACEs	.316	.058	<.001 (<.001)	.179	.052	.001 (.001)
ACEs × AUD PGS	.003	.015	.830 (.830)	−.049	.015	.001 (.002)
*Cannabis*						
CanUD PGS	.039	.076	.608 (.767)	.030	.052	.568 (.682)
ACEs	.239	.072	.001 (.003)	.403	.063	<.001 (<.001)
ACEs × CanUD PGS	−.019	.017	.260 (.520)	−.040	.017	.020 (.040)
*Opioid*						
OUD PGS	.087	.148	.554 (.767)	−.125	.097	.197 (.296)
ACEs	.097	.149	.518 (.767)	.049	.077	.523 (.682)
ACEs × OUD PGS	−.007	.028	.793 (.830)	−.008	.020	.665 (.829)
*Factor covariances*						
Alcohol & cannabis	.359	.017	<.001 (<.001)	.215	.013	<.001 (<.001)
Opioids & cannabis	.144	.033	<.001 (<.001)	.093	.015	<.001 (<.001)
Alcohol & opioids	.068	.032	.033 (.079)	.022	.015	.136 (.233)

*Note:* Models adjusted for first 10 genetic ancestry principal components, sex, age, and the interactions of sex and age with ACEs and PGS. AUD, alcohol use disorder; ACEs, adverse childhood experiences; PGS, polygenic scores; CanUD, cannabis use disorder; OUD, opioid use disorder; β, standardized coefficient; SE, standard error. *The FDR adjusted p-value is shown in parentheses.

Among EUR individuals, there were no associations between either the OUD PGS or the ACEs factor with latent OUD severity. There were no gene-by-environment interactions with OUD severity in either ancestry group. Among EUR individuals, there was no association of PGS and CanUD severity. However, in EUR participants, the ACEs factor score was positively associated with CanUD severity (b = 0.403, SE = 0.063, p < 0.001), and there was a negative gene-by-environment interaction (b = −0.040, SE = 0.015, p = 0.020). A Johnson–Neyman plot of the interaction showed that the association between ACEs and CanUD severity weakened as CanUD PGS increased and became nonsignificant among those with the highest PGS (Z 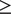 3.75; Figure 2b). Among AFR individuals, there was an association of the ACEs factor (b = 0.239, SE = 0.072, p = 0.001) with CanUD severity.

### Sensitivity analyses

Including unexposed individuals in the SUD severity models yielded stronger associations of PGS and ACEs with SUD severity than the main analyses (Supplementary Tables 4–10). In summary, in both AFR and EUR individuals, the AUD and CanUD PGS were associated with AUD and CanUD severity, respectively. ACEs were associated with all three SUD severity factors in both ancestry groups. In EUR individuals, there were also negative interactions between ACEs and PGS for all three SUD severity factors.

## Discussion

Examining PGS and ACEs in a large, diverse sample, we found *rGE* between ACEs and SUDs, as well as gene-by-environment interactions between ACEs and SUD PGS on substance use initiation and SUD severity. One possibility for the observed *rGE* is that people who inherit genetic variants associated with SUD risk may be more likely to be exposed to adverse childhood environments (Jaffee & Price, 2007) – i.e., passive r*GE.* In addition to passing down the contributory genetic variants, parents’ substance use can adversely shape family life and processes, which in turn influence children’s experiences and future substance use (Leonard & Eiden, 2007). Negative circumstances, including ACEs and substance use, often cluster together within families, likely resulting from a combination of genetics and within-family environmental influences (Holt, Buckley, & Whelan, 2008; Schwartz, Wright, & Valgardson, 2019). Importantly, the presence of r*GE* suggests that ACEs are not fully independent of SUD genetic liability.

Examining substance use initiation, we found that ACEs were associated with having ever used all three substances across both ancestries. In sensitivity analyses, ACEs were more strongly and consistently associated with SUD severity when we included individuals unexposed to substance use in the models, with the enhanced association likely driven by the robust link of ACEs with substance use initiation. PGS and ACEs interacted in association with opioid use initiation, such that the association of ACEs with initiation was reduced as PGS increased. This suggests that the impact of early life adversity on substance use initiation diminishes among those with higher genetic liability for SUDs.

ACEs were also associated with latent SUD severity, supporting previous research showing that ACEs are implicated in both the initiation of substance use and the severity of SUDs (Deol et al., 2023; Dube et al., 2006; Hines et al., 2023; Meadows et al., 2023). The strength of the associations varied by substance and ancestry group. ACEs were associated with AUD and CanUD severity in both ancestries, suggesting a more robust link to alcohol and cannabis than OUD. ACEs may increase risk for substance use by influencing emotional dysregulation, which contributes broadly to internalizing/externalizing symptoms and disorders (Evans, Goff, Upchurch, & Grella, 2020; Garland, Reese, Bedford, & Baker, 2019; Gerhardt et al., 2023; Khoury, Tang, Bradley, Cubells, & Ressler, 2010; Müller et al., 2015; Rambau et al., 2018; Sreenivasulu, Prathyusha, Ezhumalai, Narayanan, & Murthy, 2024; Tang, Ports, Zhang, & Lin, 2020; Tasmim, Le Foll, & Hassan, 2022). Overall, ACEs were more consistently and strongly associated with SUDs than the respective PGS, consistent with prior observations in this sample (Na, Deak, Kranzler, Pietrzak, & Gelernter, 2024).

We also found gene-by-environment (i.e., PGS by ACEs) interactions, the directionality of which was consistently negative, such that the impact of ACEs on AUD and CanUD severity was significant except among EUR individuals with higher than average SUD PGS. These findings are consistent with previous analyses in the Yale-Penn sample, including a study in which genetic liability for multiple SUDs similarly moderated the relationship between ACEs and SUD development (Kranzler et al., 2024). We extended these prior findings by identifying substance-specific patterns of association, whereby gene-by-environment interactions were more robust for AUD and CanUD than OUD. Interactions between genetics and ACEs in the same direction have also been previously seen for internalizing and externalizing symptomology (Wright & Schwartz, 2021).

The interactions between PGS and ACEs observed in this study highlight the importance of considering both genetic and environmental factors in the etiology of SUDs (Karcher et al., 2019). Childhood adversity may shape key pathways that increase vulnerability to substance use problems, particularly through substance use initiation. For example, early-life adversity can impair emotional regulation (Miu et al., 2022), disrupt stress-response systems (al’Absi, Ginty, & Lovallo, 2021), and alter reward processing (Oltean, Șoflău, Miu, & Szentágotai-Tătar, 2023), all of which affect substance use behaviors. Individuals exposed to childhood adversity may benefit from trauma-informed care that addresses emotional regulation skills and adaptive coping strategies, while those with high genetic liability may respond better to interventions targeting impulsivity or reward system dysregulation, biological systems implicated in genetic studies of SUDs (Deak & Johnson, 2021; Hatoum et al., 2022). Although it has not yet been shown definitively for SUDs, those with greater genetic risk may also show a greater likelihood of response to pharmacological interventions, similar to what has been demonstrated for the effect of statins on cardiovascular risk (Damask et al., 2020). In contrast, we speculate that the effects of ACEs may be more amenable to psychosocial treatments. Tailoring interventions to the specific pathways through which genetic and environmental influences manifest – interpersonal difficulties, emotional dysregulation, or impaired functioning – may improve treatment outcomes and reduce the likelihood of relapse in individuals with SUDs.

### Limitations

This study has several limitations. First, the AFR GWAS used to calculate PGS were conducted in smaller samples than the EUR GWAS, which limited power to identify associations in AFR individuals across all three substances. To date, GWAS participants have predominantly comprised EUR individuals (86.55% in 2023), and there remains a clear need for participant representation to align more closely with population demographics (Mills & Rahal, 2020). Of the substances examined, the OUD GWAS sample size was smaller than both the AUD and CanUD samples. In addition, the prevalence of OUD in the Yale-Penn sample was lower than that of either AUD or CanUD. Thus, the power to detect associations with OUD was more limited than with AUD or CanUD, which may explain the less robust findings for OUD. Although higher ACEs factor scores reflect exposure to more extensive or varied forms of childhood trauma, we could not directly examine the severity of ACEs. Thus, this warrants further study using more refined measures of trauma exposure. Finally, the SSADDA relies on self-report (in the context of a semistructured interview conducted by trained interviewers), which may be subject to erroneous recall, particularly for past substance-related behaviors and early childhood experiences.

## Conclusions

This study highlights the associations of PGS and ACEs with substance use initiation and SUD severity, including gene-by-environment interactions that vary by substance and genetic ancestry. ACEs emerged as a consistent risk factor across all substances, underscoring their broad association with substance use behaviors. Although the associations of PGS were more limited, the AUD PGS was associated with AUD severity in both EUR and AFR individuals. Gene-by-environment interactions were consistent with early life adversity having a diminished impact on opioid use initiation and SUD severity as genetic liability increased. Collectively, these findings suggest a central role for ACEs in the pathogenesis of SUDs, potentially through their relationship to the onset of substance use and progression to problematic use.

## Supporting information

SooHoo et al. supplementary material SooHoo et al. supplementary material

## Supplementary material

The supplementary material for this article can be found at http://doi.org/10.1017/S0033291725001163.
