# Supplementary material for: Associations of childhood adversity and substance use disorder polygenic scores with disorder severity and diagnostic criteria
Source: Psychol Med. 2025 May 2;55:e132. doi: 10.1017/S0033291725001163 (PMC12094658; doi:10.1017/S0033291725001163)
Supplement: SooHoo et al. supplementary material [file S0033291725001163sup001.docx]

**Supplementary Materials**

**Supplementary Table 1: CFA of Adverse Childhood Events (ACE) Model**

|  | **AFR** | | **EUR** | |
| --- | --- | --- | --- | --- |
| **Item** | **Loading*** | **Std Err** | **Loading*** | **Std Err** |
| Violent Crime | .64 | .05 | .62 | .03 |
| Sexual Abuse | .72 | .05 | .63 | .03 |
| Physical Abuse | .78 | .06 | .72 | .03 |
| 3+ Main Caregivers | .46 | .09 | .51 | .04 |
| 2+ Relocations | .42 | .06 | .35 | .03 |
| Household Substance Use | .35 | .06 | .40 | .03 |
| Household Smoking | .24 | .07 | .23 | .03 |
| No Religious Participation | .18 | .09 | .17 | .04 |
| Poor Caregiver Relationship | .68 | .07 | .69 | .03 |
| Infrequent Contact with Relatives | .31 | .09 | .13 | .05 |
| Substance Use with Smoking^ | .52 | .06 | .54 | .02 |
| **Fit Indices**  Chi-Square/df  CFA  RMSEA  SRMR | 1.04  .997  .008  .057 |  | 4.55  .954  .030  .059 |  |

* Standardized loadings, ^correlation between residuals

**Supplemental Table 2: Discovery Samples for GWAS**

| **Study** | **Cohorts** | **Study design** | **Sample size** | **Phenotype** |
| --- | --- | --- | --- | --- |
| **Zhou et al., 2023 *Nat Med* (PMID: 38062264)** |  | **Case-control and continuous trait** | **113,325/639,923** | **AUD (ICD AUD diagnosis or DSM-IV alcohol dependence)** |
|  | MVP | Case-control | 80,028/363,113 | ICD AUD diagnosis |
|  | FinnGen | Case-control | 8,866/209,926 | ICD AUD diagnosis |
|  | PGC | Case-control | 9,938/30,992 | DSM-IV alcohol dependence |
|  | QIMR Australian Genetics of Depression Study | Case-control | 6,726/4,467 | DSM-IV alcohol dependence |
|  | QIMR Twins | Case-control | 2,772/5,630 | DSM-IV alcohol dependence |
|  | QIMR Genetics of Bipolar Disorder Study | Case-control | 1,287/751 | DSM-IV alcohol dependence |
|  | iPSYCH1 | Case-control | 2,117/13,238 | DSM-IV alcohol dependence |
|  | iPSYCH2 | Case-control | 1,024/5,732 | DSM-IV alcohol dependence |
|  | Yale-Penn 3 (excluded) | Case-control | 567/1,074 | DSM-IV alcohol dependence |
| **Levey et al., 2023 *Nat Genet* (PMID: 37985822)** |  | **Case-control** | **60,765/865,653** | **ICD CanUD diagnosis** |
|  | Psychiatric Genetics Consortium (PGC)+deCODE | Case-control | 18,370/304,838 | ICD CanUD diagnosis |
|  | iPSYCH2 | Case-control | 4,733/15,560 | ICD CanUD diagnosis |
|  | Mass General Brigham (MGB) | Case-control | 456/24,088 | ICD CanUD diagnosis |
|  | MVP | Case-control | 37,206/521,167 | ICD CanUD diagnosis |
| **Kember et al., 2022 *Nat Neurosci* (PMID: 36171425 )** | **MVP** | **Case-control** | **31,473/394,471** | **ICD OUD diagnosis** |

| **Supplementary Table 3. Endorsement of substance use disorder diagnostic criteria.** | | | | | | | | | |
| --- | --- | --- | --- | --- | --- | --- | --- | --- | --- |
|  | **Cannabis** | | | **Alcohol** | | | **Opioid** | | |
|  | AFR  (N = 3515) | EUR  (N = 4,113) | p-value | AFR  (N = 4,283) | EUR  (N = 4,969) | p-value | AFR  (N = 1,628) | EUR  (N = 2,810) | p-value |
| **Criterion** |  |  |  |  |  |  |  |  |  |
| Time spent using/obtaining | 48.0% | 46.7% | .313 | 41.5% | 40.3% | .289 | 70.1% | 80.9% | <.001 |
| Continued use despite physical/psychological problems | 27.4% | 26.7% | .572 | 45.3% | 42.2% | .006 | 71.5% | 81.6% | <.001 |
| Unsuccessful efforts to decrease use | 46.3% | 36.5% | <.001 | 69.1% | 57.0% | <.001 | 78.8% | 83.9% | <.001 |
| Using more than intended | 43.7% | 41.1% | .044 | 74.3% | 69.8% | <.001 | 70.7% | 80.3% | <.001 |
| Tolerance | 34.4% | 41.3% | <.001 | 55.9% | 56.8% | .404 | 66.9% | 81.7% | <.001 |
| Withdrawal | 32.3% | 32.3% | 1.000 | 42.0% | 46.7% | <.001 | 76.3% | 85.3% | <.001 |
| Reduce other activities | 30.5% | 32.1% | .187 | 49.2% | 45.9% | .003 | 63.5% | 73.7% | <.001 |
| Failure to fulfill obligations | 24.4% | 25.7% | .236 | 48.7% | 50.0% | .246 | 56.5% | 69.1% | <.001 |
| Use in hazardous situations | 34.9% | 49.0% | <.001 | 56.1% | 66.4% | <.001 | 50.9% | 72.2% | <.001 |
| Continued Use despite social/interpersonal problems | 35.7% | 36.7% | .420 | 64.5% | 62.3% | .039 | 69.3% | 78.6% | <.001 |
| Craving | 31.2% | 28.6% | .033 | 33.4% | 32.5% | .363 | 60.3% | 77.9% | <.001 |
| *Note:* N represents the number of individuals who endorsed using each substance within each ancestry group. AFR = African-like, EUR = European-like. | | | | | | | | | |

| **Supplementary Table 4. Endorsement of individual substance use disorder criteria.** | | | | | | |
| --- | --- | --- | --- | --- | --- | --- |
|  | **Cannabis** | | **Alcohol** | | **Opioid** | |
| **Criteria** | **AFR** | **EUR** | **AFR** | **EUR** | **AFR** | **EUR** |
| Time Spent Obtaining/Using | 62.9% | 36.1% | 40.1% | 39.6% | 19.9% | 42.3% |
| Continued Use Despite Physical/Psychological Problems | 20.8% | 20.9% | 43.2% | 41.7% | 20.1% | 42.8% |
| Unsuccessful Effort to Decrease Use | 35.3% | 28.3% | 66.9% | 55.9% | 22.4% | 44.0% |
| Used More Than Intended | 33.7% | 31.8% | 72.4% | 68.2% | 19.8% | 42.1% |
| Tolerance | 26.0% | 31.9% | 53.9% | 55.7% | 18.8% | 42.8% |
| Withdrawal | 24.3% | 24.9% | 39.8% | 45.6% | 21.6% | 44.6% |
| Reduction In Other Activities | 23.0% | 24.9% | 47.2% | 45.1% | 17.7% | 38.5% |
| Failure to Fulfill Obligations | 18.4% | 19.8% | 46.9% | 49.2% | 15.6% | 36.0% |
| Used In Hazardous Situations | 26.3% | 38.4% | 54.5% | 65.4% | 14.0% | 37.5% |
| Continued Use Despite Social/Interpersonal Problems | 27.0% | 28.7% | 62.4% | 61.6% | 19.4% | 41.1% |
| Craving | 23.8% | 21.9% | 33.0% | 32.5% | 17.2% | 40.7% |
| *Note:* AFR = African-like ancestry, EUR = European-like ancestry. | | | | | | |

| **Supplementary Table 5. Results of multivariate regression models.** | | | | | | |
| --- | --- | --- | --- | --- | --- | --- |
|  | **AFR** | | | **EUR** | | |
|  | **β** | **SE** | **P-Value*** | **β** | **SE** | **P-Value*** |
| *Cannabis*  PGS  ACE  ACE*PGS | .04  .33  -.01 | .01  .01  .01 | .002  <.001  .60 | .02  .32  -.03 | .02  .01  .01 | .07  <.001  .02 |
| *Alcohol*  PGS  ACE  ACE*PGS | .06  .32  -.001 | .02  .01  .01 | <.001  <.001  .95 | .09  .33  -.04 | .01  .01  .01 | <.001  <.001  .001 |
| *Opioid*  PGS  ACE  ACE*PGS | .02  .16  -.005 | .02  .02  .02 | .28  <.001  .76 | .05  .33  -.05 | .05  .01  .01 | .32  <.001  <.001 |
| *Factor Correlations*  Cannabis & Alcohol  Cannabis & Opioids  Alcohol & Opioids | .38  .20  .13 | .02  .02  .02 | <.001  <.001  <.001 | .35  .35  .20 | .01  .01  .01 | <.001  <.001  <.001 |
| *Note:* Models adjusted for sex, age, and first 10 ancestry principal components. ACE = adverse childhood experiences, PGS = polygenic scores, AFR = African ancestry, EUR = European ancestry, β = standardized coefficient, SE = standard error. * FDR adjusted p-value for multiple testing. | | | | | | |

**Supplementary Table 6: Logistic Regression Models Results for Cannabis Criteria Items**

|  | **AFR** | | | | | **EUR** | | | | |
| --- | --- | --- | --- | --- | --- | --- | --- | --- | --- | --- |
| **Criteria** | **β** | **SE** | **OR** | **95 % CI** | **P-Val*** | **Β** | **SE** | **OR** | **95 % CI** | **P-Val*** |
| Obtaining/Using  PGS  ACE  PGS*ACE | .09  .41  -.05 | .04  .04  .04 | 1.10  1.50  0.95 | 1.02 – 1.18  1.40 – 1.61  0.89 – 1.02 | .04  <.001  .23 | .18  .32  -.04 | .04  .04  .03 | 1.20  1.38  0.96 | 1.10 – 1.31  1.29 – 1.48  0.90 – 1.03 | <.001  <.001  .24 |
| Physical/Psychiatric Problem  PGS  ACE  PGS*ACE | .06  .46  -.01 | .05  .04  .04 | 1.07  1.58  0.99 | 0.98 – 1.16  1.47 – 1.71  0.92 – 1.07 | .22  <.001  .76 | .11  .30  -.05 | .05  .04  .04 | 1.11  1.36  0.95 | 1.01 – 1.23  1.26 – 1.46  0.89 – 1.02 | .04  <.001  .19 |
| Decrease Use  PGS  ACE  PGS*ACE | .02  .27  -.07 | .04  .04  .04 | 1.02  1.31  0.94 | 0.95 – 1.10  1.22 – 1.40  0.87 – 1.00 | .56  <.001  .13 | .14  .32  -.09 | .05  .04  .03 | 1.15  1.37  0.91 | 1.06 – 1.26  1.28 – 1.47  0.85 – 0.97 | .002  <.001  .01 |
| More than Intended  PGS  ACE  PGS*ACE | .07  .37  -.03 | .04  .04  .04 | 1.07  1.45  0.97 | 0.99 – 1.15  1.35 – 1.56  0.90 – 1.04 | .15  <.001  .40 | .19  .34  -.05 | .04  .04  .03 | 1.21  1.40  0.95 | 1.11 – 1.32  1.30 – 1.50  0.89 – 1.02 | <.001  <.001  .14 |
| Tolerance  PGS  ACE  PGS*ACE | .10  .33  -.06 | .04  .04  .04 | 1.11  1.39  0.94 | 1.02 – 1.20  1.30 – 1.50  0.88 – 1.01 | .04  <.001  .18 | .12  .34  -.07 | .05  .04  .03 | 1.13  1.41  0.94 | 1.03 – 1.23  1.31 – 1.51  0.88 – 1.00 | .01  <.001  .06 |
| Withdrawal  PGS  ACE  PGS*ACE | .06  .37  -.07 | .04  .04  .04 | 1.06  1.45  0.94 | 0.98 – 1.15  1.35 – 1.56  0.87 – 1.01 | .21  <.001  .15 | .19  .34  -.10 | .05  .04  .03 | 1.21  1.41  0.91 | 1.10 – 1.33  1.31 – 1.51  0.85 – 0.97 | <.001  <.001  .01 |
| Reduce Activities  PGS  ACE  PGS*ACE | .07  .34  -.04 | .04  .04  .04 | 1.07  1.40  0.96 | 0.99 – 1.16  1.30 – 1.51  0.89 – 1.03 | .18  <.001  .32 | .13  .30  -.07 | .05  .04  .03 | 1.14  1.35  0.93 | 1.04 – 1.25  1.26 – 1.45  0.87 – 1.00 | .01  <.001  .05 |
| Fulfill Obligations  PGS  ACE  PGS*ACE | .04  .34  -.06 | .05  .04  .04 | 1.04  1.41  0.94 | 0.95 – 1.14  1.30 – 1.53  0.87 – 1.02 | .42  <.001  .20 | .18  .31  -.11 | .05  .04  .04 | 1.20  1.33  0.91 | 1.09 – 1.32  1.23 – 1.44  0.85 – 0.98 | .001  <.001  .01 |
| Hazardous Situation  PGS  ACE  PGS*ACE | .06  .33  -.03 | .04  .04  .04 | 1.06  1.39  0.98 | 0.98 – 1.14  1.29 – 1.50  0.91 – 1.05 | .23  <.001  .54 | .15  .31  -.11 | .04  .04  .03 | 1.16  1.36  0.90 | 1.07 – 1.26  1.27 – 1.46  0.84 – 0.96 | .001  <.001  .002 |
| Social/Interpersonal  PGS  ACE  PGS*ACE | .06  .36  -.04 | .04  .04  .04 | 1.06  1.43  0.96 | 0.98 – 1.15  1.33 – 1.53  0.89 – 1.03 | .21  <.001  .32 | .09  .35  -.13 | .05  .04  .03 | 1.09  1.41  0.88 | 1.00 – 1.19  1.32 – 1.52  0.83 – 0.94 | .06  <.001  .001 |
| Craving  PGS  ACE  PGS*ACE | .08  .39  -.05 | .04  .04  .04 | 1.08  1.47  0.95 | 1.00 – 1.18  1.37 – 1.59  0.88 – 1.02 | .13  <.001  .23 | .10  .31  -.07 | .05  .04  .04 | 1.10  1.37  0.93 | 1.00 – 1.21  1.27 – 1.47  0.87 – 1.00 | .06  <.001  .048 |

Models adjusted for sex, age, and first 10 ancestry principal components. β = standardized regression coefficient, SE = standard error, OR = odds ratio, AFR = African Ancestry, EUR = European ancestry.

* FDR adjusted p-value for multiple testing

**Supplementary Table 7: Logistic Regression Models Results for Alcohol Criteria Items**

|  | **AFR** | | | | | **EUR** | | | | |
| --- | --- | --- | --- | --- | --- | --- | --- | --- | --- | --- |
| **Criteria** | **β** | **SE** | **OR** | **95 % CI** | **P-Val*** | **Β** | **SE** | **OR** | **95 % CI** | **P-Val*** |
| Obtaining/Using  PGS  ACE  PGS*ACE | .09  .27  .02 | .04  .03  .03 | 1.10  1.31  1.02 | 1.02 – 1.18  1.23 – 1.40  0.95 – 1.08 | .03  <.001  .74 | .16  .32  -.06 | .03  .03  .03 | 1.18  1.37  0.94 | 1.10 – 1.26  1.29 – 1.46  0.88 – 1.00 | <.001  <.001  .08 |
| Physical/Psychiatric Problem  PGS  ACE  PGS*ACE | .01  .38  -.01 | .04  .04  .03 | 1.01  1.47  0.99 | 0.94 – 1.09  1.37 – 1.57  0.93 – 1.06 | .77  <.001  .87 | .18  .42  -.09 | .03  .03  .03 | 1.20  1.52  0.92 | 1.12 – 1.28  1.42 – 1.62  0.86 – 0.98 | <.001  <.001  .02 |
| Decrease Use  PGS  ACE  PGS*ACE | .14  .29  -.05 | .04  .04  .04 | 1.14  1.33  0.95 | 1.06 – 1.23  1.24 – 1.44  0.89 – 1.03 | .001  <.001  .32 | .24  .33  -.05 | .03  .04  .04 | 1.27  1.39  0.94 | 1.18 – 1.35  1.29 – 1.49  0.87 – 1.01 | <.001  <.001  .09 |
| More than Intended  PGS  ACE  PGS*ACE | .11  .39  -.04 | .04  .04  .04 | 1.12  1.48  0.96 | 1.03 – 1.21  1.36 – 1.61  0.89 – 1.05 | .02  <.001  .50 | .23  .32  -.07 | .04  .04  .04 | 1.25  1.55  0.96 | 1.17 – 1.35  1.42 – 1.68  0.88 – 1. 04 | <.001  <.001  .36 |
| Tolerance  PGS  ACE  PGS*ACE | .03  .23  .04 | .04  .03  .03 | 1.03  1.26  1.04 | 0.96 – 1.11  1.18 – 1.35  0.97 – 1.11 | .50  <.001  .33 | .20  .31  .004 | .03  .04  .04 | 1.22  1.36  1.00 | 1.14 – 1.30  1.27 – 1.46  0.94 – 1.08 | <.001  <.001  .92 |
| Withdrawal  PGS  ACE  PGS*ACE | .03  .30  -.05 | .04  .04  .04 | 1.03  1.35  0.95 | 0.96 – 1.12  1.26 – 1.45  0.89 – 1.02 | .50  <.001  .32 | .16  .35  -.04 | .04  .04  .04 | 1.17  1.42  0.96 | 1.09 – 1.26  1.32 – 1.52  0.90 – 1.03 | <.001  <.001  .34 |
| Reduce Activities  PGS  ACE  PGS*ACE | .05  .35  .05 | .04  .04  .03 | 1.05  1.42  1.05 | 0.97 – 1.12  1.33 – 1.53  0.98 – 1.12 | .32  <.001  .26 | .26  .32  -.07 | .03  .03  .03 | 1.30  1.38  0.93 | 1.22 – 1.39  1.29 – 1.47  0.87 – 0.99 | <.001  <.001  .045 |
| Fulfill Obligations  PGS  ACE  PGS*ACE | .09  .36  -.01 | .04  .04  .03 | 1.10  1.43  0.99 | 1.02 – 1.18  1.33 – 1.53  0.93 – 1.06 | .03  <.001  .91 | .21  .36  -.03 | .03  .03  .04 | 1.23  1.44  0.97 | 1.15 – 1.32  1.35 – 1.54  0.91 – 1.04 | <.001  <.001  .42 |
| Hazardous Situation  PGS  ACE  PGS*ACE | .09  .33  .02 | .04  .04  .04 | 1.10  1.39  1.02 | 1.02 – 1.18  1.30 – 1.49  0.96 – 1.10 | .03  <.001  .61 | .24  .44  -.07 | .04  .04  .04 | 1.27  1.55  0.93 | 1.18 – 1.36  1.43 – 1.67  0.86 – 1.01 | <.001  <.001  .10 |
| Social/Interpersonal  PGS  ACE  PGS*ACE | .09  .44  .001 | .04  .04  .04 | 1.09  1.55  1.00 | 1.01 – 1.17  1.43 – 1.67  0.93 – 1.08 | .05  <.001  .98 | .25  .49  -.07 | .04  .04  .04 | 1.29  1.62  0.93 | 1.20 – 1.38  1.50 – 1.76  0.86 – 1.01 | <.001  <.001  .09 |
| Craving  PGS  ACE  PGS*ACE | .06  .25  -.02 | .04  .03  .03 | 1.06  1.28  0.98 | 0.98 – 1.14  1.20 – 1.37  0.92 – 1.05 | .25  <.001  .61 | .18  .26  -.05 | .04  .03  .03 | 1.20  1.30  0.95 | 1.12 – 1.28  122 – 1.39  0.89 – 1.01 | <.001  <.001  .12 |
|  |  |  |  |  |  |  |  |  |  |  |

Models adjusted for sex, age, and first 10 ancestry principal components. β = standardized regression coefficient, SE = standard error, OR = odds ratio, AFR = African Ancestry, EUR = European ancestry.

* FDR adjusted p-value for multiple testing

**Supplementary Table 8: Logistic Regression Models Results for Opioid Criteria Items**

|  | **AFR** | | | | | **EUR** | | | | |
| --- | --- | --- | --- | --- | --- | --- | --- | --- | --- | --- |
| **Criteria** | **β** | **SE** | **OR** | **95 % CI** | **P-Val*** | **Β** | **SE** | **OR** | **95 % CI** | **P-Val*** |
| Obtaining/Using  PGS  ACE  PGS*ACE | .03  .26  -.05 | .05  .04  .04 | 1.041.290.95 | 0.94 – 1.14  1.19 – 1.40  0.89 – 1.03 | .51  <.001  .33 | .28  .44  -.08 | .14  .04  .03 | 1.321.550.92 | 1.01 – 1.73  1.44 – 1.66  0.87 – 0.98 | .06  <.001  .01 |
| Physical/Psychiatric Problem  PGS  ACE  PGS*ACE | .05  .26  -.02 | .05  .04  .04 | 1.051.290.98 | 0.96 – 1.16  1.19 – 1.40  0.91 – 1.05 | .38  <.001  .54 | .18  .49  -.09 | .14  .04  .03 | 1.191.630.92 | 0.91 – 1.56  1.51 – 1.74  0.86 – 0.97 | .23  <.001  .01 |
| Decrease Use  PGS  ACE  PGS*ACE | .05  .22  -.04 | .05  .04  .04 | 1.051.250.96 | 0.96 – 1.15  1.15 – 1.35  0.90 – 1.04 | .38  <.001  0.96 | .30  .47  -.10 | .14  .04  .03 | 1.341.600.90 | 1.03 – 1.76  1.49 – 1.72  0.85 – 0.96 | .047  <.001  .002 |
| More Intended  PGS  ACE  PGS*ACE | .07  .26  -.03 | .05  .04  .04 | 1.081.300.97 | 0.98 – 1.18  1.20 – 1.41  0.91 – 1.05 | .24  <.001  .51 | .15  .50  -.06 | .14  .04  .03 | 1.161.640.94 | 0.89 – 1.52  1.53 – 1.76  0.89 – 1.00 | .29  <.001  .07 |
| Tolerance  PGS  ACE  PGS*ACE | .05  .27  -.03 | .05  .04  .04 | 1.051.310.97 | 0.96 – 1.16  1.20 – 1.42  0.90 – 1.05 | .38  <.001  .51 | .20  .44  -.10 | .14  .04  .03 | 1.221.550.91 | 0.94 – 1.60  1.44 – 1.66  0.86 – 0.96 | .17  <.001  .003 |
| Withdrawal  PGS  ACE  PGS*ACE | .05  .25  -.04 | .05  .04  .04 | 1.051.280.96 | 0.95 – 1.15  1.18 – 1.39  0.89 – 1.03 | .40  <.001  .38 | .23  .49  -.09 | .14  .04  .03 | 1.261.630.91 | 0.96 – 1.65  1.52 – 1.76  0.86 – 0.97 | .11  <.001  .01 |
| Reduce Activities  PGS  ACE  PGS*ACE | .08  .31  -.06 | .05  .04  .04 | 1.091.360.94 | 0.98 – 1.20  1.25 – 1.48  0.87 – 1.02 | .21  <.001  .24 | .08  .43  -.08 | .14  .04  .03 | 1.091.540.92 | 0.83 – 1.42  1.44 – 1.65  0.87 – 0.98 | .57  <.001  .01 |
| Fulfill Obligations  PGS  ACE  PGS*ACE | .09  .28  -.05 | .05  .05  .04 | 1.091.330.95 | 0.98 – 1.21  1.22 – 1.45  0.88 – 1.03 | .21  <.001  .33 | .08  .44  -.09 | .14  .04  .03 | 1.081.550.91 | 0.82 – 1.42  1.44 – 1.66  0.86 – 0.97 | .55  <.001  .01 |
| Hazardous Situation  PGS  ACE  PGS*ACE | .10  .26  -.11 | .06  .05  .04 | 1.101.290.90 | 0.99 – 1.23  1.18 – 1.42  0.83 – 0.98 | .19  <.001  .04 | .28  .42  -.11 | .14  .04  .03 | 1.321.520.89 | 1.00 – 1.73  1.46 – 1.63  0.84 – 0.95 | .06  <.001  <.001 |
| Social/Interpersonal  PGS  ACE  PGS*ACE | .10  .28  -.05 | .05  .04  .04 | 1.101.320.95 | 1.00 – 1.21  1.21 – 1.43  0.88 – 1.02 | .12  <.001  .28 | .16  .45  -.07 | .14  .04  .03 | 1.171.570.94 | 0.89 – 1.53  1.46 – 1.68  0.88 – 0.99 | .28  <.001  .045 |
| Craving  PGS  ACE  PGS*ACE | .04  .24  -.05 | .05  .04  .04 | 1.041.270.96 | 0.94 – 1.15  1.17 – 1.38  0.88 – 1.03 | .51  <.001  .38 | .29  .41  -.09 | .14  .04  .03 | 1.331.510.92 | 1.02 – 1.74  1.41 – 1.62  0.86 – 0.97 | .053  <.001  .01 |

Models adjusted for sex, age, and first 10 ancestry principal components. β = standardized regression coefficient, SE = standard error, OR = odds ratio, AFR = African Ancestry, EUR = European ancestry.

* FDR adjusted p-value for multiple testing

| **Supplementary Table 9: Logistic Regression Model Results for Use in AFR** | | | | |
| --- | --- | --- | --- | --- |
| **Model** | **Estimate** | **SE** | **Statistic** | **P Value** |
| *Opioid Use* |  |  |  |  |
| OUD PGS | 0.07 | 0.04 | 1.79 | 0.07 |
| ACES | 0.33 | 0.03 | 10.09 | 6.01 E-24 |
| OUD PGS X ACES | -0.03 | 0.03 | -1.06 | 0.29 |
| *Alcohol Use* |  |  |  |  |
| AUD PGS | 0.10 | 0.14 | 0.74 | 0.46 |
| ACES | 0.84 | 0.17 | 5.00 | 5.83 E-07 |
| AUD_PGS X ACES | -0.02 | 0.16 | -0.15 | 0.88 |
| *Cannabis Use* |  |  |  |  |
| CanUD PGS | 0.04 | 0.05 | 0.86 | 0.39 |
| ACES | 0.73 | 0.06 | 13.12 | 2.54 E-39 |
| CanUD PGS X ACES | 0.02 | 0.06 | 0.40 | 0.69 |

| **Supplementary Table 10: Logistic Regression Model Results for Use in EUR** | | | | |
| --- | --- | --- | --- | --- |
| **Model** | **Estimate** | **SE** | **Statistic** | **P-value** |
| *Opioid Use* |  |  |  |  |
| OUD PGS | 0.24 | 0.13 | 1.81 | 0.07 |
| ACES | 0.58 | 0.04 | 14.93 | 2.23 E-50 |
| OUD PGS X ACES | -0.11 | 0.03 | -3.56 | <0.001 |
| *Alcohol Use* |  |  |  |  |
| AUD PGS | 0.212 | 0.15 | 1.43 | 0.15 |
| ACES | 0.92 | 0.21 | 4.30 | 1.72 E-05 |
| AUD PGS X ACES | -0.09 | 0.20 | -0.44 | 0.66 |
| *Cannabis Use* |  |  |  |  |
| CanUD PGS | 0.32 | 0.05 | 5.85 | 4.86 E-09 |
| ACES | 0.59 | 0.06 | 10.20 | 1.91 E-24 |
| CanUD PGS X ACES | -0.05 | 0.06 | -0.91 | 0.36 |
